# Hypomethylation of intron1 of α-synuclein gene does not correlate with Parkinson’s disease

**DOI:** 10.1186/s13041-017-0285-z

**Published:** 2017-02-07

**Authors:** Subhrangshu Guhathakurta, Baggio A. Evangelista, Susmita Ghosh, Sambuddha Basu, Yoon-Seong Kim

**Affiliations:** 1 Division of Neurosciences, Burnett School of Biomedical Sciences, University of Central Florida College of Medicine, 6900 Lake Nona Blvd, Orlando, FL 32827 USA; 2 Kyunghee University Medical College, Seoul, Korea

**Keywords:** Epigenetic regulation, DNA methylation, Parkinson’s Disease, α-synuclein

## Abstract

Deregulation of α-synuclein encoding gene (*SNCA*) is one of the important facets of Parkinson’s disease (PD) research. DNA methylation status of *SNCA*-intron1 has been shown to regulate the α-synuclein expression. The present study is aimed at investigating whether methylation of *SNCA*-intron1 is associated with higher expression of α-synuclein in PD. We have investigated the intron1 methylation status from 16 post-mortem brain samples comprised of 8 PD and 8 control subjects using bisulfite sequencing. We further correlated this methylation status with α-synuclein protein levels in substantia nigra of that individual using western blot analysis. We did not observe any significant difference in methylation of *SNCA*-intron1 region between PD and control samples. Moreover, no correlation was observed between methylation of *SNCA*-intron1 with α-synuclein level. Methylation of *SNCA*-intron1 region does not correlate with α-synuclein expression in PD samples.

## Introduction

Role of α-synuclein (α-SYN) in the pathogenesis of Parkinson′s disease (PD) is undeniable. Epigenetic regulation of α-SYN encoding gene (*SNCA*) has been greatly explored focusing on the methylation status of intron1 CpG island [1–9]. *SNCA* harbors 6 exons of which two upstream exons (1A and 1B) remain non-coding and part of its 5′ untranslated region [1]. This intron1 region right before the first coding exon (exon 2) has been shown to regulate gene expression to a great extent by differential methylation of its CpG island and also by recruitment of several transcription factors [1, 10]. It is known that *SNCA* duplication/triplication is strongly associated with familial form of PD and this gene multiplication has also been shown to produce significantly higher mRNA in the cell [11–13]. Thus it can be conferred that higher expression of α-SYN can lead to PD pathogenesis. Since hypomethylation of CpGs of a gene-regulatory region is generally associated with increased expression of the gene, it is hypothesized that decrease in methylation in the intron1 of *SNCA* might increase expression of α-SYN in PD [1]. In the present study, we have investigated the methylation status of the *SNCA*-intron1 in the *substantia nigra* of post-mortem PD patients and matched controls to decipher the association of DNA methylation in this region and PD pathogenesis. Moreover, we have also correlated this methylation status with the level of α-SYN in the subjects.

## Methods

### Post-mortem brain samples

In the present study, 16 post-mortem brain samples were investigated which consisted of 8 PD and 8 control subjects. The 7 samples from each group were obtained from NIH Neurobio bank consortium. Age ranged from 73 to 83 years (average 78.71 years) and post-mortem interval (PMI) varied from 6.7 hours to 15 hours (average 11.67 hours) in PD cases. Similarly, the age of the controls ranged from 54 years to 89 years with an average of 73.53 years. PMI for the controls varied from 10 hours to 30.25 hours (average 24.02 hours). One control and PD brain sample were procured from UK Brain bank. Age and post-mortem delay information for those two subjects were not available to us.

### *SNCA* methylation analysis

**Fig. 1:**
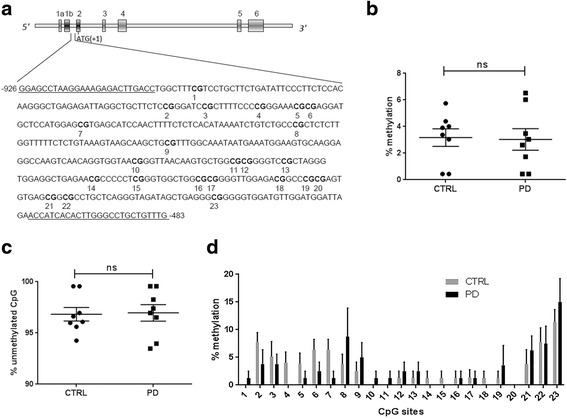
Methylation of *SNCA*-intron1 does not differ between PD and controls. *SNCA* contains 6 exons. A 444 bp region in the intron1 (-483 to -926 bp with respect to ATG) was investigated. The sequence of the studied region is shown and the 23 CpGs are marked by numbers. The priming regions are underlined (**a**). Relative level of methylation (**b**) and unmethylated CpG (**c**) between control (CTRL) and PD groups are shown. No significant difference was observed between two groups for mean methylation level. Analysis of individual CpG site was done from control (*n* = 8) and PD (*n* = 8) subjects (**d**). Analysis did not reveal any significant difference between the groups. For every subject, 9 to 10 clones were studied to get the mean methylation percentage. The data is represented as mean ± SEM. Pair-wise comparison was made by Mann-Whitney t-test to analyze the significance. n.s. represents non-significant difference

Around 25 mg of SN tissue from each freshly frozen sample was precisely isolated by punch biopsy. DNA was extracted using Quick-DNA Universal Kit (Zymo Research; Catalogue No. D4068). Around 500 nanograms of DNA per sample was used for sodium bisulfite conversion using EZ DNA methylation kit (Zymo Research; Catalogue No. D5001) with little modifications following their optimization guide to ensure complete bisulfite conversion. Each reaction was made in duplicate to increase the amount of template DNA for the PCR. To amplify the intron1 region of *SNCA* spanning 23 CpG sites, we used the primers as described by Jowaed et al. [1]. EpiMark Hot Start Taq DNA polymerase (NEB Inc; Catalogue No. M0490S) was used for the PCR amplification. The amplified PCR products of 444 base pair (Fig.1a) were then cloned into pGEM-T Easy vector (Promega; Catalogue No. A137A) and 9 to 10 positive colonies per PCR product were sequenced using T7 promoter or SP6 reverse primers.

### Western blot analysis

Protein level of α-SYN from each sample was analyzed using 25-30 mg of freshly frozen SN tissue. Lysis was done in 100 μL of RIPA buffer (Radio Immuno Precipitation Assay buffer; 1% NP-40; 0.5% Sodium deoxycholate; 0.1% SDS, supplemented with protease inhibitor) at 4 °C. Equal amount of protein was loaded for all the samples in a 12% SDS-polyacrylamide gel and the separated proteins were then transferred onto a nitrocellulose membrane. The α-SYN and β-actin protein bands were detected using specific primary antibodies (α-SYN, BD Transduction Laboratories catalogue No. 610787, dilution 1: 500 and β-actin, Sigma, Catalogue No. A5316, dilution: 1:10,000). An anti-mouse HRP (Horse radish peroxidase)-conjugated secondary antibody (Jackson Immuno Research, dilution 1:5,000) was used to visualize the bands by enhanced chemiluminescent technique. Since, the two samples from UK brain bank were collected in 2011, there was not enough protein samples available at the time of study. Therefore, those two samples were not included for the studies on α-SYN protein level.

### Statistical analysis

To calculate the relative level of methylation between PD and control subjects, QUMA software was used for the analysis with criteria that filters out the clonal PCR sequences and analyses only unique clones of the sample [14]. To determine the amount of methylation for each individual at 23 different CpG sites, percentage methylation of each of the CpG site of those 9 to 10 clones were calculated and averaged. Non-parametric Mann-Whitney t-test was applied to assess the significant difference in the mean methylation level between control and PD. Difference in normalized α-SYN expression (α-SYN/β-actin) between the groups was measured using Mann-Whitney t-test. To determine the correlation between percentage-demethylation with α-SYN expression, non-parametric Spearman′s Rank correlation was used for the groups followed by linear regression analysis. All the statistical analyses and graphical representations were done using GraphPad Prism software version 5.0. Significance was assessed at 95% level. Data are presented as mean ± SEM.

## Results

**Fig. 2:**
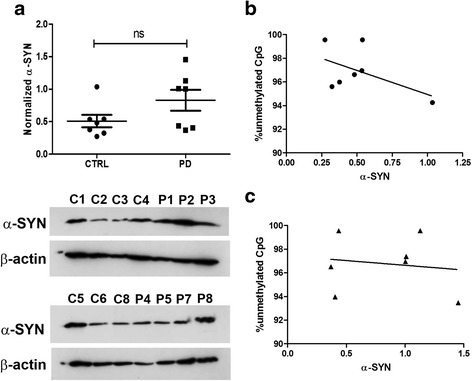
Methylation status of *SNCA*-intron1 does not correlate with α-SYN level. Total α-SYN level was measured for both control (*n* = 7) and PD (*n* = 7) groups using western blot analysis. The gel picture shows the level of α-SYN and β-actin in each samples, where C1 to C8 represent controls and P1 to P8 represent the PD subjects respectively. The α-SYN level was compared between both the groups after normalizing it to respective β-actin. No significant difference in α-SYN levels was observed between control and PD using Mann-Whitney t-test (**a**). Correlation between percentage of demethylation with α-SYN levels for control (**b**) and PD patients (**c**) were carried out. The test did not reveal any significant correlation in any group with α-SYN levels. Spearman’s rank correlation analysis was applied in both the groups. n.s. represents non-significant difference

In the present study, *SNCA*-intron1 region is comparably hypomethylated both in control (3.17 ± 0.66%) and PD (3.04 ± 0.81%) samples, and there is no significant difference in the methylation level between the groups (*p* = 0.9) (Fig.1b and c). We have also explored the mean methylation of individual CpG site (Fig. 1a and d). Pair-wise comparison of each CpG also did not reveal any significant difference in methylation between PD and control (Fig. 1d). The α-SYN protein level, although higher in PD cases, did not demonstrate any significant difference when compared to controls (*p* = 0.26) (Fig. 2a). At the same time, we did not observe any significant correlation between amount of unmethylated CpG and α-SYN expression in any group (*r* = -0.20, *p* = 0.67 control and *r* = 0.05, *p* = 0.92 PD) (Fig. 2b and c).

In the present cohort of samples, we did not find any significant correlation between either age (*r* = 0.39, *p* = 0.39 for controls; *r* = 0.10, *p* = 0.83 for PD) or PMI (*r* = -0.07, *p* = 0.89 for controls; *r* = 0.25, *p* = 0.59 for PD) of the study subjects with respective methylation status using Spearman′s rank correlation test. It was also previously shown by de Boni et al., that there is no correlation between PMI and methylation in Lewy Body disease (LBD) cases or in control [3]. However, they found a slightly significant correlation with age of the LBD cases and methylation [3].

## Discussion

Regulation of *SNCA* expression by its methylation status of inton1 has been widely studied in relation to PD [1–9]. It′s important to note that *SNCA* like any other gene has several regulatory regions and variations, which have been shown to regulate this gene′s expression significantly irrespective of methylation status of *SNCA*-intron1 [15–17]. However, it’s also interesting that *SNCA*- intron1 methylation varies widely in different type of cells and demethylation of this region is positively correlated with higher expression of α-SYN [1, 2]. Therefore, it is important to study *SNCA*-intron1 methylation status in the SN of PD and control to understand if hypomethylation of this gene can be correlated with the disease.

In the present report, we have explored the methylation status of *SNCA*-intron1 in the SN of post-mortem PD and control subjects. As reported by others [1, 3, 6, 7], we also have observed that mean methylation of this locus in the SN is extremely low (Fig. 1c). We have studied a region of intron1 which was previously studied by Jowaed et al. [1], encompassing 23 CpG sites (Fig. 1a). We did not find any significant hypomethylation in PD subjects as compared to the controls (Fig. 1c). No association of *SNCA*-intron1 methylation in PD subjects was previously reported by other groups as well [3, 5, 7]. However, several groups reported significant difference in methylation between PD and control [1, 2, 4, 6, 8, 9]. This apparent difference in the outcome could be attributed to several factors like investigation of different CpGs regions in different studies, admixture of different cell types in the SN region apart from dopaminergic neurons and also may be due to difference in the sample characterization. Previously it was shown using luciferase reporter assay, that a significant demethylation of *SNCA*-intron1 could lead to an increase in α-SYN expression as compared to the completely methylated one [1]. Similarly, HEK293 cells treated with dopamine demonstrated induction of a sizable amount of demethylation (94.4% to 21.2% methylation) in this region and increased α-SYN expression [2]. Most of the studies on human brain samples including the present one have reported that *SNCA*-intron1 region in the SN is significantly hypomethylated (≥90%) both in control and PD which might be responsible for the constitutive expression of α-SYN in both groups. So, it can be envisaged that a small difference in methylation of *SNCA*-intron1 between PD and control, might have a limited effect on further expression of α-SYN [8]. Since, methylation of individual CpG site also can play a role in transcription factor binding [1, 3, 6–8], we have examined all 23 CpG sites separately in both the groups but failed to find any significant difference (Fig. 1d). However, we observed that 2^nd^ to 7^th^ CpG sites have a trend of hypomethylation in PD (Fig. 1d). Two other groups found significant differences in some of the CpG sites in PD cases which they hypothesized to play a significant role in increased transcription of *SNCA* [1, 8]. On the other hand, another study couldn’t find any site-specific hypomethylation in intron1 when they studied different brain regions, instead they found hypermethylation of few CpGs in some tissues in specific stages of LBD (Table 1) [3]. Some studies reported a difference in intron1 methylation between PD and controls from PBMC (peripheral blood mononuclear cells), however, some studies failed to find any such difference [5–8]. It has been already shown that methylation status varies between tissues and not necessarily mimics the situation in brain cell types [1, 2, 5–9].

**Table 1:** List of different studies investigated on *SNCA*-intron1 methylation

Author group	Tissues studied	Number	CpG sites studied in intron1	Method	Site specific change in methylation as compared to control	Overall *SNCA*-intron-1 methylation	Correlation with α-SYN
Jowaed et. al., 2010 [1]	Post-mortem brain samples	SNpc and cortex (*n* = 6 PD, *n* = 6 controls), putamen (*n* = 6 PD, *n* = 8 control)	23 CpG sites	Bisulfite/Sanger sequencing	SNpc and Putamen, hypomethylated	Hypomethylated in SNpc, cortex and putamen	Not studied
Matsumoto et al., 2010 [2]	Post-mortem brain samples	SN (*n* = 3 PD, *n* = 1 DLB, *n* = 3 control) , anterior cingulate cortex (*n* = 12 PD/DLB, *n* = 8 control), putamen (*n* = 7 PD/DLB, *n* = 4 control)	13 CpG sites (10^th^ to 22^nd^ as compared to ours)	Bisulfite/Sanger sequencing	Not studied	Hypomethylated only in SN	Not studied
De Boni et al., 2011 [3]	Post-mortem brain samples	SN (*n* = 10 LBD, *n* = 3 control), putamen (*n* = 15 LBD, control *n* = 6), cingulate gyrus (*n* = 15 LBD, *n* = 6 controls), temporal cortex (*n* = 15 LBD, *n* = 6 controls), cerebellum (*n* = 14 LBD, *n* = 6 controls)	19 CpG sites (2^nd^ to 22^nd^ (excepting 13^th^ and 18^th^ as compared to ours)	Bisulfite/next-generation sequencing	Putamen, hypermethylated in limbic and neocortical stages of LBD	Hypermethylated in putamen in case of limbic stage of LBD. Other tissues no significant change	Not studied
Desplats et al., 2011 [4]	Post-mortem brain samples	Frontal cortex (*n* = 4 PD, *n* = 4 DLB, *n* = 4 control)	Gross methylation of Intron1 region	Methylation sensitive PCR	Not studied	Hypomethylation	Not studied
Richter et al., 2012 [5]	PBMC	Idiopathic PD (*n* = 43), monogenic PD (*n* = 3), controls (*n* = 37)	7 CpG sites (16^th^ to 22^nd^ as compared to ours)	Bisulfite/pyrosequencing	Not studied	No significant change	Not studied
Tan et al., 2014 [6]	PBMC	PD (*n* = 50), control (*n* = 49)	14 CpG sites (10^th^ to 23^rd^ as compared to ours)	Bisulfite/Sanger sequencing	hypomethylated	Hypomethylated	α-SYN expression increased with decreased methylation of intron1 in a small representative group of subjects
Song et al., 2014 [7]	PBMC	PD (*n* = 50), control (*n* = 50)	13 CpG sites (10^th^ to 22^nd^ as compared to ours)	Bisulfite/pyrosequencing	Non-significant decrease	No significant difference	Not studied
Ai et al., 2014 [8]	PBMC	PD (*n* = 100), control (*n* = 95)	23 CpG sites	Bisulfite/Sanger sequencing	hypomethylated	Hypomethylation	No difference in α-SYN expression between PD and control
Pihlstrom et al., 2015 [9]	PBMC and post-mortem brain samples	PBMC (*n* = 36 PD, *n* = 36 control), cortex (*n* = 12 PD, *n* = 12 control)	Gross methylation of Intron1 region	Methylation sensitive restriction digestion and quantitative PCR	Not studied	Hypomethylation in PBMC but no significant difference in post-mortem brain tissue	No difference in α-SYN expression between PD and control

*PD* Parkinson’s disease, *SNpc* Substantia nigra pars compacta, *LBD* Lewy body diseases, *PBMC* Peripheral blood mononuclear cells, Number of subjects are only related to *SNCA*-intron1 methylation studies

Concurrently, we have also investigated α-SYN levels in these two groups but as expected, failed to find any significant difference in the protein level (Fig. 2a). However, both groups contained high as well as low α-SYN expressing subjects. This overall non-significant difference in protein levels between the groups might be partially explained by the observed non-significant difference in DNA methylation. One group showed a positive correlation between decreased intron1 methylation with increased α-SYN expression in PD cases [6]. However, two other studies reported no difference in α-SYN expression between PD and controls but they found a significant hypomethylation in intron1 [8, 9]. This further points out a limited effect of intron1 methylation on overall α-SYN level or transcription.

Together, our study demonstrates a lack of association between *SNCA*-intron1 methylation and PD. However, this study points out the importance of studying a comprehensive epigenetic regulation of α-SYN rather than focusing only on DNA methylation status of this gene.

## Abbreviations

HEK293: Human Embryonic Kidney 293T immortalized cell line
PD: Parkinson’s disease
PMI: Post-mortem interval
SN: Substantia Nigra
*SNCA*: Synuclein, Alpha (Non A4 Component Of Amyloid Precursor); Alpha-synuclein encoding gene
α-SYN: alpha-synuclein
